# Journal of Orthopaedic Surgery and Research Editorial

**DOI:** 10.1186/1749-799X-1-1

**Published:** 2006-09-25

**Authors:** Cheng-Kung Cheng, Kai-Ming Chan

**Affiliations:** 1 Orthopaedic Biomechanics Laboratory, Institute of Biomedical Engineering, National Yang Ming University, Taipei, Taiwan; 2 Department of Orthopaedics & Traumatology, The Chinese University of Hong Kong, Hong Kong, China

## Abstract

*Journal of Orthopaedic Surgery and Research *is an open access, online journal that aims to expeditiously publish clinical and basic research studies related to musculoskeletal issues in different cultural communities. It provides a platform for exchanges of new clinical and scientific information in the most precise and expeditious way to achieve dissemination of information and cross-fertilization of ideas. The open access nature of this journal allows articles to be universally and freely accessible via the Internet. This ensures a rapid and efficient communication of research findings. The journal welcomes all types of articles related to musculoskeletal issues. Its timely publication and high visibility are the two most important features that make this journal different from other traditional journals in the orthopaedic field.

## Editorial

### Aims

*Journal of Orthopaedic Surgery and Research *is an open access, online journal that aims to expeditiously publish clinical and basic research studies related to musculoskeletal issues in different cultural communities.

## Field description

Orthopaedic research has been conducted essentially at clinical and basic science levels. With advancement of new technologies and increasing demand and aspiration from the community, we are seeing an enormous growth of orthopaedic research, in particular in the fields of traumatology, spinal surgery, joint replacement, sports medicine, musculoskeletal tumour, hand and micro-surgery, foot and ankle surgery, paediatric orthopaedic, and orthopaedic rehabilitation. The contribution of basic science ranges from molecular, cellular, structural, and functional perspectives to tissue engineering, gait analysis, automation and robotic surgery. Implant and biomaterial designs are emerging new disciplines that will compliment clinical applications. Multi-disciplinary research with collaboration amongst clinicians and scientists from different disciplines will be the trend in the coming decades. This journal will provide the platform for exchanges of new clinical and scientific information in the most precise and expeditious way to achieve timely dissemination of information and cross-fertilization of ideas.

## Rationale

This new journal will provide the incentive and facilitation for academic exchanges from different cultural, social, economical and ethnic perspectives, allowing unique orthopaedic research in different communities and cultures to be shared around the world. With a professional editorial team [1] and facilities of translation, we shall present quality research in the international language of English to reach a wide readership amongst professionals working in different disciplines on musculoskeletal issues. We also aim at collaboration amongst academic institutions, research facilities of industries and corporate to promote integration for new technology and clinical applications.

## Open access

*Journal of Orthopaedic Surgery and Research *welcomes all types of articles related to musculoskeletal issues, which include Research, Reviews, Book reviews, Case reports, Case studies, Commentaries, Database articles, Debate articles, Hypotheses, Meeting reports, Methodology articles, Short reports, Software articles and Study protocols. In order to cover all the essential topics in the orthopaedic field, *Journal of Orthopaedic Surgery and Research *will publish issues with different themes, examples include Spinal Arthroplasty, Implant Design, Biomaterial, Functional Tendinopathies, and Scoliosis.

*Journal of Orthopaedic Surgery and Research *is an online open access journal and as such articles are universally and freely accessible to everyone in an easily readable format via the Internet. The copyright belongs to the authors and they grant anyone the right to reproduce and disseminate the article on condition that the article has been correctly cited and no errors have been introduced [2]. Articles submitted to *Journal of Orthopaedic Surgery and Research *will be published quickly continuously online once accepted after the peer-review process avoiding the delays commonly found in traditional journals, in which submitted and accepted papers may have to wait for publication until enough articles have been collected for a particular issue. Articles from *Journal of Orthopaedic Surgery and Research *are not only included in BioMed Central's databases, but also permanently archived in PubMed Central, the US National Library of Medicine's full-text repository of life science literature, in repositories at the University of Potsdam in Germany, at INIST in France and in e-Depot, the National Library of the Netherlands' digital archive of all electronic publications.

Being an open access journal ensures rapid and efficient communication of research findings for the benefits of science, medicine and the general public. Authors can be assured their work is disseminated to the widest possible audience. Information will not be limited to readers' library's budgets, which in return will enhance literature searching of the articles. Results of publicly funded research will be available to all, including those without access to a library with a subscription. Resource-poor countries and institutions will be able to access the same material as wealthier ones.

Timely publication and high visibility of *Journal of Orthopaedic Surgery and Research *are two important features of this journal that makes it different from other traditional journals in the orthopaedic field.

## Peer review policy

Three reviewers will be assigned for each submission. The reviewers will have up to three weeks to review the submitted article. After the receipt of the recommendation from the reviewers, the decision on whether to Accept, Reject or request Major/Minor Revisions will be made by co-editors-in-chief. The review time will be monitored by the Editorial Board to make it reasonably short for this journal.
